# Does the intensity of dissociation predict antidepressant effects 24 hours after infusion of racemic ketamine or esketamine in treatment-resistant depression? A secondary analysis from a randomized controlled trial

**DOI:** 10.47626/2237-6089-2022-0593

**Published:** 2025-04-07

**Authors:** Mariana V. F. Echegaray, Rodrigo P. Mello, Guilherme M. Magnavita, Gustavo C. Leal, Fernanda S. Correia-Melo, Ana Paula Jesus-Nunes, Flávia Vieira, Igor D. Bandeira, Ana Teresa Caliman-Fontes, Manuela Telles, Lívia N. F. Guerreiro-Costa, Roberta Ferrari Marback, Breno Souza-Marques, Daniel H. Lins-Silva, Cassio Santos-Lima, Taiane de Azevedo Cardoso, Flávio Kapczinski, Acioly L. T. Lacerda, Lucas C. Quarantini

**Affiliations:** 1 Universidade Federal da Bahia Hospital Universitário Professor Edgard Santos Laboratório de Neuropsicofarmacologia, Serviço de Psiquiatria Salvador BA Brazil Laboratório de Neuropsicofarmacologia, Serviço de Psiquiatria, Hospital Universitário Professor Edgard Santos, Universidade Federal da Bahia (UFBA), Salvador, BA, Brazil.; 2 UFBA Faculdade de Medicina da Bahia Salvador BA Brazil Programa de Pós-Graduação em Medicina e Saúde, Faculdade de Medicina da Bahia, UFBA, Salvador, BA, Brazil.; 3 UFBA Instituto de Psicologia Salvador BA Brazil Programa de Pós-Graduação em Psicologia, Instituto de Psicologia, UFBA, Salvador, BA, Brazil.; 4 McMaster University Department of Psychiatry and Behavioral Neurosciences Hamilton Canada Department of Psychiatry and Behavioral Neurosciences, McMaster University, Hamilton, Canada.; 5 Universidade Federal de São Paulo Laboratório Interdisciplinar de Neurociências Clínicas São Paulo SP Brazil Laboratório Interdisciplinar de Neurociências Clínicas, Universidade Federal de São Paulo, São Paulo, SP, Brazil.; 6 Instituto Sinapse de Neurociências Clínicas Campinas SP Brazil Instituto Sinapse de Neurociências Clínicas, Campinas, SP, Brazil.; 7 UFBA Faculdade de Medicina da Bahia Departamento de Neurociências e Saúde Mental Salvador BA Brazil Departamento de Neurociências e Saúde Mental, Faculdade de Medicina da Bahia, UFBA, Salvador, BA, Brazil.

**Keywords:** Ketamine, esketamine, treatment-resistant depression, major depressive disorder, dissociation

## Abstract

**Objective:**

Ketamine and esketamine have both shown significant antidepressant effects in treatment-resistant depression (TRD) and conflicting evidence suggests that dissociation induced by these drugs could be a clinical predictor of esketamine/ketamine's efficacy.

**Methods:**

This study is a secondary analysis of data from a two-center, randomized, controlled trial. Participants were randomly assigned 1:1 to receive an IV infusion of either esketamine (0.25 mg/kg) or racemic ketamine (0.50 mg/kg) over 40 minutes. Dissociative symptoms were assessed using the Clinician-Administered Dissociative State Scale (CADSS) 40 minutes following the beginning of the infusion. Variations in depression scores were measured with the Montgomery-Åsberg Depression Rating Scale (MADRS), which was administered before the intervention as a baseline measure and 24 hours, 72 hours, and 7 days following infusion.

**Results:**

Sixty-one patients were included in the analysis. Examining CADSS scores of 15 or below, for every 1-point increment in the CADSS score, there was a mean change of −0.5 (standard deviation [SD] = 0.25; p = 0.04) of predicted MADRS score from baseline to 24 hours. The results for 72 hours and 7 days following infusion were not significant. Since the original trial was not designed to assess the relationship between ketamine or esketamine-induced dissociation and antidepressant effects as the main outcome, confounding variables for this relationship were not controlled.

**Conclusion:**

We suggest a positive relationship between dissociation intensity measured with the CADSS and the antidepressant effects of ketamine and esketamine 24 hours after infusion for CADSS scores of up to 15 points.

## Introduction

Major depressive disorder (MDD) is a recurrent and disabling psychiatric condition, and it is the single biggest contributor to non-fatal health loss worldwide.^1^ The main goal of MDD treatment is to achieve remission, which translates to patients returning to their previous level of functioning.^2^ Approximately one-third of patients with MDD fail to achieve remission with available treatments, which is associated with a poor prognosis in clinical and functional terms.^3-6^ Over recent decades, new strategies for managing treatment-resistant depression (TRD) have been proposed. N-methyl-D-aspartate (NMDA) antagonists, such as ketamine and its enantiomers, S (+)-ketamine (esketamine) and R (-)-ketamine (arketamine), have demonstrated a robust antidepressant effect and acceptable tolerability in the short term.^7-12^ Recently, the Canadian Network for Mood and Anxiety Treatments (CANMAT) guideline included a single infusion of racemic ketamine as having level 1 evidence for treatment of TRD,^13^ and the Food and Drug Administration (FDA) also approved intranasal esketamine for TRD.^14^ Identifying predictors of response to the effects of ketamine or esketamine could facilitate patient selection for more intensive regimes, therefore decreasing expenditure on futile care and reducing side effects.^15^ A series of predictors are being tested, but results have not yet proved conclusive.^16^ Although potentially less precise, clinical predictors may represent a cheaper and simpler strategy for maximizing the effects of ketamine and esketamine.^17^ The validity of dissociation as a clinical predictor of antidepressant efficacy has been extensively studied, with mixed results.^6,17-20^ A recent systematic review addressed this matter and concluded that further clarification is needed since two out of five studies found a significant correlation between depression scores and induced dissociation as measured by the Clinician-Administered Dissociative State Scale (CADSS).^21^ Moreover, only one publication examined the relationship between esketamine induced dissociation and antidepressant effects.^22^

The aim of this study was to assess the relationship between dissociation induced with racemic ketamine or esketamine and their antidepressant effects 24 hours, 72 hours, and 7 days following infusion in TRD subjects. We hypothesized that a higher intensity of dissociative side effects would predict improvement of depressive symptoms. We performed a post-hoc analysis of data from the first head-to-head study between ketamine and esketamine.^9^

## Methods

### Study design and location

This study is a secondary analysis of data from a randomized, active-controlled, double-blinded trial with two parallel groups conducted at Universidade Federal da Bahia (UFBA) and Universidade Federal de São Paulo (UNIFESP), both located in Brazil.^9^

### Ethical considerations

The study was approved by the local Institutional Review Boards (Hospital Universitário Professor Edgard Santos: 46657415.0.0000.0049; Hospital São Paulo: 46657415.0.3001.5505) and adheres to the ethical principles of the Declaration of Helsinki, 2013. The study protocol was registered with the Japan Primary Registries Network (JPRN): UMIN000032355. Additional information is available in the previously published protocol.^23^

### Participants

We included participants over 18 years of age with an MDD diagnosis based on the Diagnostic and Statistical Manual of Mental Disorders, 4th edition (DSM-IV) criteria and confirmed by experienced psychiatrists and psychologists using the Brazilian version of the Mini International Neuropsychiatric Interview 5.0.0 (MINI- Plus).^24^ All included patients were diagnosed with TRD, which is defined as therapeutic failure after at least one adequate antidepressant treatment lasting for a minimum of 12 weeks, considering that there is no consensual definition of TRD and some studies classify this as its first stage.^25^

Exclusion criteria were: (a) concomitant treatment with electroconvulsive therapy; (b) diagnosis of a psychotic disorder; (c) intellectual disability or dementia; (d) unstable heart disease; and (e) current illicit drug use. In addition to using a less restrictive definition of TRD, we did not exclude other psychiatric or clinical comorbidities or augmentation treatments for TRD, in order to obtain a naturalistic sample.

Patients maintained their previous treatment regimen (types of medications and doses remaining unchanged) for at least 15 days before randomization and were unable to change dosages or start to use new drugs during the study follow-up week (except for non-benzodiazepine sleep inducers). All participants voluntarily agreed to participate and signed a written Informed Consent Form.

### Intervention

Each participant received a single dose of one of the two drugs in the study: ketamine racemic mixture (0.5 mg/kg) or esketamine (0.25 mg/kg). Drugs were infused intravenously for 40 minutes.

### Randomization

Participants were randomized into esketamine and ketamine groups, on a 1:1 ratio, using electronic randomization software (http://www.randomizer.org). Randomization was carried out by a single independent investigator for both locations. The only professionals who knew which drug had been infused were the investigator responsible for the allocation and two nurses responsible for drug preparation (one for each center). These professionals did not participate in any clinical evaluation.

### Outcomes and assessment

Dissociative symptoms were assessed using the 23-item CADSS^26^ at the 40th minute of drug infusion. CADSS is the most widely used scale for assessment of dissociative symptoms induced by ketamine or esketamine.^27-29^ Depression severity was assessed using Montgomery-Åsberg Depression Rating Scale (MADRS)^30^ scores. The MADRS was administered before the intervention as a baseline measure and 24 hours, 72 hours, and 7 days following infusion.

### Statistical analyses

Statistical analyses were conducted using IBM SPSS v. 25 for Windows and the R statistical package).^31^ The normality of samples was determined by Q-Q plot and histogram analysis. Descriptive statistics are presented as frequencies, means, and medians based on variable distribution. We used Student's *t* tests for univariate comparisons of group means, Mann-Whitney *U* tests for univariate comparisons of group medians, and Pearson's chi-squared test or Fisher's exact test for univariate comparisons of proportions.

We calculated the absolute variation in the MADRS scores between baseline and the three post-infusion measurement points at 24 hours, 72 hours, and 7 days. We then used a Locally Weighted Scatterplot Smoothing (LOESS) curve for each variation and the CADSS scores, allowing us to visually assess non-linear relationships between antidepressant response and dissociation. These curves seemed to indicate a non-linear relationship resembling two straight lines with a break at 15 points on the CADSS. We then fitted a longitudinal fixed-effects model with time, treatment group, and CADSS scores as predictors. Fixed models are akin to mixed models, and they work by modeling the response variable – in our cases the MADRS score – as a function of time and any number of specified covariates. Their main advantage is the parsimony of assumptions when the outcome is measured at fixed time intervals, as we did. By using an unstructured covariance matrix, we are able to control for observation interdependence and calculate p-values while making few assumptions regarding the behavior of covariance over time. To better model the non-linear CADSS x MADRS relationship, we compared four different types of modeling strategies: a linear model, an exponential model, a quadratic model, and different piecewise linear splines with a single knot at different CADSS values. We selected a piecewise spline with a knot at 15 points on the CADSS scale on the basis of model fit, Akaike information criterion (AIC), likelihood ratio tests, and p-values for the coefficients. All longitudinal models in the final report were estimated using restricted maximum likelihood (REML).

We conducted all analyses with interaction terms allowing the ketamine and esketamine groups to be treated as separate from one another, to allow for effect modification of the CADSS x MADRS relationship by type of ketamine. Since these terms did not reveal different profiles for the types of ketamine, in relation to p-value, model fit, or AIC, we decided to drop the interactions and treat both ketamine groups as similar. These findings are in line with results from the non-inferiority study, which showed no significant differences between these two forms of ketamine in terms of efficacy or safety outcomes.

## Results

The complete clinical outcomes of the trial have been published previously.^9^ Sixty-three patients were included in the original study, but CADSS scores were incomplete for two of these patients, so this secondary analysis only includes 61 of them. Of these patients, 32 received esketamine infusion and 29 received ketamine. There were no statistically significant differences between the two groups for the observed variables. Table 1 shows the participants’ characteristics at baseline. Almost all of the participants in the esketamine group (90.6%) and all of the participants in the ketamine group had proved resistant to at least two previous antidepressant treatments.

**Table 1:** Baseline sociodemographic and clinical characteristics of the participants

Characteristics		Esketamine group n (%)	Ketamine group n (%)	p-value
Total sample		32 (52.5)	29 (47.5)	
Female gender		18 (56.3)	20 (69.0)	0.30*
				
Ethnicity				0.47*
	White	11 (34.3)	13 (44.8)	
	Black	5 (15.6)	6 (20.7)	
	Mixed ethnicity	16 (50.0)	10 (34.4)	
				
Income (MW)				0.16*
	Below 1	10 (31.2)	5 (17.2)	
	1 to 3	6 (18.7)	13 (44.8)	
	3 to 6	4 (12.5)	2 (6.9)	
	6 to 10	7 (21.8)	6 (20.7)	
	Above 10	5 (15.6)	3 (10.3)	
				
≥ 2 therapeutic failures		29 (90.6)	29 (100.0)	0.14†
PTSD		0 (0.0)	3 (10.3)	0.10†
GAD		21 (65.6)	19 (67.9)	0.85*
PD		10 (55.6)	8 (44.4)	0.82*
Age (years) (mean, SD)		44.5 (13.7)	48.6 (15.1)	0.27‡
MADRS at baseline (median, IQR)		31.5 (14)	32 (8)	0.96§

GAD = generalized anxiety disorder; IQR = interquartile range; MADRS = Montgomery-Åsberg Depression Rating Scale; MW = minimum wage; PD = panic disorder; PTSD = post-traumatic stress disorder; SD = standard deviation.

* Pearson's chi-square test;

† Fisher's exact test;

‡ Student's *t* test;

§ Mann-Whitney *U* test.

**Figure 1:**
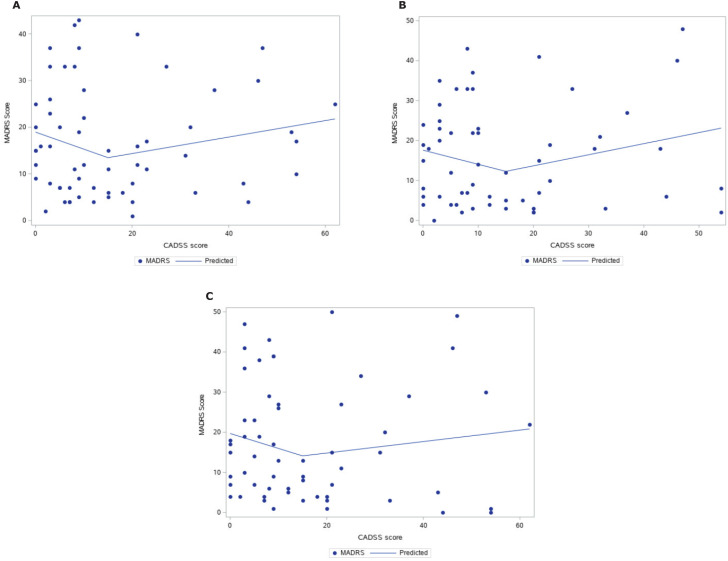
Relationship between induced dissociative symptoms and predicted depressive symptom severity after ketamine or esketamine infusion. Induced dissociative symptoms were measured with the Clinician-Administered Dissociative States Scale (CADSS) at the 40th minute of ketamine or esketamine infusion. Depressive symptom severity was measured by Montgomery-Åsberg Depression Rating Scale (MADRS) scores. Predicted depressive symptom severity was estimated with a longitudinal fixed-effects model with time (24 hours [A], 72 hours [B] or 7 days [C]), treatment group, and CADSS scores as predictors. Since these terms did not reveal different profiles for the types of ketamine, in terms of p-values, model fit, or Akaike information criterion (AIC), we decided to drop the interactions and treat both as ketamine. The model selected is a piecewise spline with a knot at 15 points on the CADSS scale, based on model fit, AIC, likelihood ratio tests, and p-values for the coefficients.

The median CADSS score was 9 (interquartile range [IQR] 16; range 0-62) for the esketamine group and 15 (IQR 26; range 0-54) for the ketamine group, with no statistical difference between the groups (p = 0.4). For CADSS scores of 15 or below, every 1-point increment in the CADSS score was associated with a mean change of −0.5 (SD = 0.25; p = 0.04) in predicted MADRS score from baseline to 24 hours. For CADSS scores greater than 15, there was a mean change of 0.01 (95% confidence interval [95%CI] −0.11 to 0.3; p = 0.3) of predicted MADRS score from baseline to 24 hours for every 1-point increase in CADSS. This relationship is illustrated in Figure 1A. The results for 72 hours (Figure 1B) and 7 days (Figure 1C) following infusion were not significant. Considering CADSS scores of 15 or below, for every 1-point increment in the CADSS score, there was a mean change of −0.5 (SD = 0.28; p = 0.07) in the predicted MADRS score from baseline to 72 hours and a mean change of −0.5 (SD = 0.31; p = 0.10) in the predicted MADRS score from baseline to 7 days.

## Discussion

The findings of the present study suggest that the intensity of dissociative symptoms induced by both racemic ketamine and esketamine is associated with the antidepressant effects observed 24 hours after infusion. This relationship was similar in the ketamine and esketamine groups. Although induced dissociation may be a clinical marker of the antidepressant effect of ketamine and esketamine, this evidence was restricted to those with CADSS scores up to 15. To our knowledge, this is the first study to identify a specific cutoff point for the relationship between dissociation and the changes in depressive symptoms induced by different enantiomeric forms of ketamine.

Luckenbaugh et al.^19^ carried out a study with 108 patients diagnosed with TRD or bipolar disorder to evaluate possible predictors of antidepressant efficacy after ketamine use (0.5 mg/kg by intravenous infusion over 40 minutes). They showed a significant association between increased CADSS scores at 40 minutes and antidepressant efficacy measured by Hamilton Depression Rating Scale (HDRS) scores at 230 minutes and 7 days, but not at 24 hours. Niciu et al.^32^ extended these findings by examining specific CADSS subscale scores (depersonalization, derealization, and amnesia) for 126 patients suffering from a major depressive episode (both unipolar and bipolar disorders), who also received a single (0.5 mg/kg intravenous [i.v.]) ketamine infusion. The results demonstrated that depersonalization was positively related to antidepressant effects. It should be noted, however, that both studies have limitations in respect to the highly heterogeneous population. Most recently, Phillips et al.^20^ assessed dissociation after infusion of 0.5 mg/kg of ketamine in 22 participants with TRD. They found a significant association between antidepressant response and variation on CADSS scores at 24 hours post-infusion. Based on an analysis of data from a phase 3, randomized withdrawal (maintenance-of-effect) study of intranasal esketamine, an advisory committee to the US Food and Drug Administration (FDA) concluded that dissociative symptoms are associated with a delay in depression relapse. However, their analysis could not determine whether this association was due to an unblinding effect or to a direct antidepressant effect linked to dissociation.^33^

The relationship between ketamine-induced dissociation and antidepressant response is not clear, as shown by studies with contrasting results. Valentine et al.^34^ found no association between an increase in CADSS scores and antidepressant effects after one intravenous ketamine infusion (0.5 mg/kg) in 10 subjects with MDD. Lapidus et al.^35^ showed no association between dissociation intensity after administration of intranasal ketamine and antidepressant effects in 18 participants with depression who had failed at least one prior antidepressant trial. A recent study conducted with 99 participants with TRD also failed to show an association between CADSS scores and improvement in HDRS scores on day 1 and day 3.^18^ Subjects were assigned to one of five arms, either to receive a single dose of ketamine 0.1 mg/kg i.v., a single dose of ketamine 0.2 mg/kg i.v., a single dose of ketamine 0.5 mg/kg i.v., a single dose of ketamine 1 mg/kg i.v., or a single dose of midazolam 0.045 mg/kg i.v. (active placebo). Only the participants who received 0.5 mg/kg (n = 22) and 1.0 mg/kg of ketamine (n = 20) had a significant increase in CADSS scores, which could have affected the posterior analysis. A systematic review, including 17 studies of patients with depression, also did not find an association between CADDS and antidepressant response.^36^

Two more recent studies have analyzed this relationship in multiple-dose treatments. Wlodarczyk et al.^37^ found no association in a study with eight doses of intravenous ketamine (0.5 mg/kg) in 49 inpatients with TRD or bipolar disorder. Chen et al.^22^ published the first results concerning esketamine induced dissociation. They analyzed data from three Phase III trials of multiple doses of intranasal esketamine and did not find a correlation between dissociation and antidepressant effects. These aforementioned conflicting results regarding the relationship between dissociation and antidepressant action were similar in the fact that they all adopted linear statistical analysis. Since it is unlikely that the psychometric variables show a linear relationship throughout the assessment, as required by Pearson's correlation, or even a monotonic relationship, as assumed by Spearman's correlation, nonlinear exploratory analysis may prove useful in future studies in the area.

There are some possible explanations for our result. There is evidence that dissociative symptoms correlate with blood ketamine levels.^38,39^ However, the correlation between blood ketamine/esketamine levels and therapeutic efficacy may be encompassed within a therapeutic window for ketamine/esketamine that has not been defined yet.^40^ Singh et al.^11^ showed that lower doses of esketamine (0.2 mg/kg versus 0.4 mg/kg i.v.) have equivalent efficacy but better tolerability. The therapeutic windows for other drugs have been established, but initial adjustments were necessary. It is now well known that higher doses of first-generation antipsychotics only induce more extrapyramidal effects without increasing efficacy for controlling psychotic symptoms.^41^ The antidepressant response to nortriptyline also has a specific window and there is a curved relationship between blood levels and efficacy, meaning that a level that is too low or too high can compromise response.^42^ We hypothesize that a similar phenomenon may happen with ketamine/esketamine.

Another aspect to consider is the influence of the psychoactive state as an inherent part of the antidepressant effect, as is often credited to the therapeutic effects of serotonergic psychedelics such as psilocybin, lysergic acid diethylamide (LSD), dimethyltryptamine (DMT), and ayahuasca.^43^ The CADSS is designed to assess dissociation as an adverse effect, which can be experienced by some individuals in a qualitatively negative way, especially at higher levels, and this could also influence our findings. One study showed that ketamine non-responders had significantly higher scores than responders in the "anxious ego-disintegration" 5-Dimensional Altered State of Consciousness Rating Scale (5D-ASC) subscale, but another study, however, did not find a significant correlation between 5D-ASC dimensions and MADRS percentage change.^44,45^ Ketamine/esketamine can also induce other acute psychoactive effects, which are not well captured by the CADSS, such as mystical experiences,^46^ characterized by feelings of oneness, experiences of joy, sacredness or holiness, and acknowledging that the experience provides a new understanding of the reality.^47^ A study that used ketamine to treat cocaine addiction showed that mystical experiences, measured by the Hood Mysticism Scale, mediated therapeutic efficacy.^48^ In this study, the dissociative symptoms assessed by CADSS were associated with but did not mediate therapeutic efficacy.

One challenge to this relationship between dissociation and the antidepressant effect of ketamine is the possible role of R(-)-ketamine (arketamine). Animal studies^49,50^ and only one study in humans^51^ suggested that use of arketamine would produce an antidepressant effect without occurrence of psychotomimetic effects. Indeed, many ketamine or esketamine responders do not experience drug provoked dissociation.^22^ One possible explanation is that dissociative experiences or other acute psychoactive effects induced by ketamine/esketamine may have an additional antidepressant effect, as occurs with serotonergic psychedelics. Regardless, dissociation may be important as a cheap and safe clinical predictor of treatment efficacy, even if it is not important as a mediator of efficacy.

### Limitations

These findings should be interpreted with caution, as our study had several limitations. Firstly, the results presented in this study were from a secondary analysis. The original study was not designed to assess the relationship between dissociation induced by ketamine or esketamine and their antidepressant effects as the main outcome. Secondly, there may be confounding variables for this relationship that were not controlled for in this study, such as pharmacokinetic measures and personality disorders. To conduct the study in a naturalistic way, a diagnosis of a personality disorder was not an exclusion criterion in the protocol. Since this variable was not evaluated, it was not possible to control for it. Most studies do not exclude comorbid BPD and no studies have controlled pharmacokinetic measures for this relationship.

## Conclusion

Our study suggests a positive relationship between dissociation intensity, measured by CADSS, and antidepressant effect 24 hours after ketamine and esketamine infusion for patients with CADSS scores of up to 15 points. By identifying the cutoff point for the relationship between dissociation and response, this study may enable future investigators to pursue a therapeutic window for ketamine/esketamine. We suggest that further studies adjust this relationship for potential confounding variables.
